# Genome-wide meta-analysis of genetic susceptible genes for Type 2 Diabetes

**DOI:** 10.1186/1752-0509-6-S3-S16

**Published:** 2012-12-17

**Authors:** Paul J Hale, Alfredo M López-Yunez, Jake Y Chen

**Affiliations:** 1 School of Informatics, Indiana University-Purdue University, Indianapolis, IN, USA; 2 Indiana Center for Systems Biology and Personalized Medicine, Indianapolis, IN, USA; 3 Alevio Medical Center, Indianapolis, IN, USA; 4 Department of Computer & Information Science, Purdue University, Indianapolis, IN, USA; 5 Wenzhou Medical College, Wenzhou, Zhejiang Province, China

## Abstract

**Background:**

Many genetic studies, including single gene studies and Genome-wide association studies (GWAS), aim to identify risk alleles for genetic diseases such as Type II Diabetes (T2D). However, in T2D studies, there is a significant amount of the hereditary risk that cannot be simply explained by individual risk genes. There is a need for developing systems biology approaches to integrate comprehensive genetic information and provide new insight on T2D biology.

**Methods:**

We performed comprehensive integrative analysis of Single Nucleotide Polymorphisms (SNP's) individually curated from T2D GWAS results and mapped them to T2D candidate risk genes. Using protein-protein interaction data, we constructed a T2D-specific molecular interaction network consisting of T2D genetic risk genes and their interacting gene partners. We then studied the relationship between these T2D genes and curated gene sets.

**Results:**

We determined that T2D candidate risk genes are concentrated in certain parts of the genome, specifically in chromosome 20. Using the T2D genetic network, we identified highly-interconnected network "hub" genes. By incorporating T2D GWAS results, T2D pathways, and T2D genes' functional category information, we further ranked T2D risk genes, T2D-related pathways, and T2D-related functional categories. We found that highly-interconnected T2D disease network “hub” genes most highly associated to T2D genetic risks to be PI3KR1, ESR1, and ENPP1. The well-characterized TCF7L2, contractor to our expectation, was not among the highest-ranked T2D gene list. Many interacted pathways play a role in T2D genetic risks, which includes insulin signalling pathway, type II diabetes pathway, maturity onset diabetes of the young, adipocytokine signalling pathway, and pathways in cancer. We also observed significant crosstalk among T2D gene subnetworks which include insulin secretion, regulation of insulin secretion, response to peptide hormone stimulus, response to insulin stimulus, peptide secretion, glucose homeostasis, and hormone transport. Overview maps involving T2D genes, gene sets, pathways, and their interactions are all reported.

**Conclusions:**

Large-scale systems biology meta-analyses of GWAS results can improve interpretations of genetic variations and genetic risk factors. T2D genetic risks can be attributable to the summative genetic effects of many genes involved in a broad range of signalling pathways and functional networks. The framework developed for T2D studies may serve as a guide for studying other complex diseases.

## Background

Type 2 Diabetes (T2D) is a complex metabolic disease that affects 25.8 million Americans in 2011, according to statistics reported by Centers for Disease Control and Prevention (CDC). T2D occurs when the body develops resistance to insulin due to the malfunction of insulin producing β-cells. The developmental process of T2D involves a complex interplay between genetic and environmental factors. However, it is not clear how the underlying genetic defects give rise to T2D pathogenesis over time. Recent T2D genetic study results, particularly those from genome-wide association studies (GWAS), have yielded insights to the molecular mechanisms and underlying genetic risk factors of T2D [1]. Among the many risk genes identified are: transcription factor 7-like 2 (TCF7L2)[2-4], peroxisome proliferator-activated receptor gamma (PPARG)[5-7], and potassium inwardly-rectifying channel, subfamily J, member 11 (KCNJ11)[5,6].

These GWAS results were challenging to interpret. Many single nucleotide polymorphisms (SNPs) identified from GWAS tend to show strong sample biases and may not extrapolate from one population to another. In T2D, only approximately 28% of the disease heritability may be explained by identified individual SNPs that showed statistical significance in these samples/population--a problem known as missing heritability [8]. The combined effects of multiple risk SNP's can increase the overall odds ratio of T2D by 1.24 per allele for up to 8.68 among 18 risk alleles in one study [9] and by 1.265 per allele in another study [6]. The additive effect suggests the presence of molecular system structures that are essential to T2D pathogenesis.

To confirm the presence of molecular systems structures that may better explain missing heritability problems for T2D, we adopted a Systems Biology approach to studying T2D genetic risk gene networks as a whole rather than the risk genes individually. Prior to this study, several reports [10,11] examined genes implicated T2D differential expressions in affected tissues. In this study, we used T2D-associated SNP information curated from the Type 2 Diabetes Genetic Association Database (T2DGADB), which integrated comprehensively reported SNPs, their odds ratios, population description, and all related metadata from various T2D GWAS performed worldwide [12]. We further annotated individual SNPs collected from T2DGADB with information from the DbSNP database [13], including information such as nearby genes, Chromosomal location, gene functional class, and base changes. To create a model for T2D genetic risk gene molecular systems structure, we built a gene interaction network seeded by T2D risk genes collected from T2DGADB and expanded with high-confidence protein interaction data collected from the Human Annotated and Predicted Protein Interaction database (HAPPI) [14]. We also ranked risk genes in the network according to these high confidence interactions.

## Methods

### T2D risk SNPs and risk genes data collection and curation

Data from both the ftp site and web pages of T2DGADB were downloaded. On the ftp site, only a gene list and a SNP list without annotation were available for download. Therefore, the complete information from individual web pages of T2DGADB was extracted into a single Excel file manually. Data entries with dbSNP SNP cross-references were kept and entries without dbSNP SNP cross-reference information were removed from this study. Gene annotation information is derived from the VEGA [15] database. The Excel file was imported into the ORACLE 11 g database for subsequent efficient database querying.

### Statistical significance testing of T2D risk SNPs and risk genes

Once we collected information integrated from T2DGADB and dbSNP, we applied standard hyper-geometric tests (using an R software package called phyper) to the data set to determine which chromosomes were over-represented/under-represented. We determined significance on three data sets. First, the distribution of dbSNP of all human SNP's in current build of known origins were compared against that of risk SNP's across all chromosomes. Second, the distribution of genes where the risk SNPs can be mapped to were compared against that of all the genes across all chromosomes. Third, the distribution of protein-coding genes where the risk SNPs can be mapped to were compared against that of all the protein-coding genes across all chromosomes.

### Construction and analysis of the T2D risk genes network

To generate risk gene network, we incorporated protein interaction data from the HAPPI database. The database integrated protein-protein interactions comprehensively from STRING [16], OPHID [17], BIND [18], HPRD [19], and MINT [20] to generate an overall confidence score for each interaction. High-quality interactions (e.g., confidence score > 0.8 in the database version of the HAPPI) are strongly correlated with physical binding based protein interaction relationships. Using risk genes from T2DGADB as input, we queried the HAPPI database and retrieved related high-quality protein interactions involving risk genes as one of the interaction partner to build a risk gene protein interaction network. The retrieved genes in the T2D risk gene subnetwork were then ranked with the following network gene ranking method, which was originally introduced in [21]:

(1) $$r_{p}=k*\ln\left(\sum_{q\in NET}conf\left(p,q\right)\right)-\ln\left(\sum_{q\in NET}N\left(p,q\right)\right)$$

In this equation, *r_p _*score measures the relative significance of a gene/protein in the subnetwork. *p *and *q *are proteins in the subnetwork. *k *is a constant set at two for our purposes. *conf *(*p*, *q*) is the confidence score of the interaction between the two proteins provided by the HAPPI database and is 0 if *p *and *q *do not interact. N (p, q) is 1 if the proteins have an interaction and is 0 otherwise. With all of this we are able to generate a network map of all involved genes.

Using *r_p _*score as a base score that considers T2D disease context in the molecular interaction network, we further defined a modified score, *r_mod_*, to take into account of genes with strong genetic ties to T2D.

(2) $$r_{mod}=r_{p}\times \sqrt{P_{count}}\times OR_{avj}$$

*r_mod _*adjusts *r_p _*score of any risk gene with both the count of populations, *P_count_*, in which significant risk SNPs were identified, and the average odds ratio (*OR_avj_*) of reported risk SNPs found in these studies. Genes containing only one significant study can still be adjusted using the formula provided here. In constructing the final T2D risk gene network, we modified the original network to exclude studies in which the risk genes were determined to be insignificant (*r_p _*score < 2) before we calculated *r_mod _*scores for risk genes. Cytoscape software was used to visualize network relationships among genes and gene sets (to be described next).

### Construction and analysis of the T2D risk genes network

To further sift the results and explore functional connections, we also mapped genes onto known gene sets. For this purpose, we used DAVID [22,23] to search for enriched KEGG [24] pathways. We also used GARNET [25] to identify enriched Gene Ontology categories and their relationships.

## Results

### SNPs identified from various T2D GWAS

Based on T2DGADB, we collected 4358 T2D SNP entries that cover 518 PubMed articles reporting T2D Genome-wide association studies (GWAS) results worldwide. Since not all study reported statistics on all SNPs, there are only 3720 SNP entries with P-values, 2715 SNP entries with complete odds ratios, 2406 SNP entries with sample size and minor allele frequency values. All together, there are 1269 SNP entries with the above-mentioned complete set of statistics. After comparing collected information against dbSNP entries manually, we validated 333 SNP gene annotations, re-annotated 11 SNP gene annotations, and flagged 140 additional genes that do not appear to be consistent with dbSNP curated locus information. The "mis-annotations" in T2DGADB were partly due to the presence of two genes, e.g., ABCC8 and KCNJ11, for some reported SNPs, therefore confounding manual curations. In other cases, gene symbols that are similar to each other, e.g., AGER and RAGE, which do not even appear on the same chromosome, also seemed to have been mixed up. The flagging of putative genes, e.g., LOC387761, according to dbSNP was performed, because we wanted to prioritize genes with known gene functions. In the end, we collected 4085 distinct SNP entries that cover 1539 SNPs in 370 different genes. Among these SNP entries, 598 SNPs from 255 different genes passed a P-Value significance cutoff of 0.05.

### Observation of chromosomal specificity for T2D risk genes

We calculated the distribution of curated T2D SNPs and genes across different chromosomes and showed the statistic significance using P-values in Table 1. The results showed that chromosomes 1, 6, 11, 12, 17, 20 are over-represented for T2D SNPs mapped to the chromosome, with chromosome 20 being most significant (P-value = 3.3E-38). To adjust for potential over-sampling of certain well-annotated T2D genes for genotyping studies in the GWAS results, we also report the significance testing results based on the distribution of genes that contained risk SNPs (namely "all genes") and the distribution of protein-coding genes that contained risk SNPs (namely "coding genes"). The results showed that chromosomes 1, 3, 7, 19, and 20 are over-represented for SNP-mapped T2D genes, with chromosome 20 being most significant (P-value = 6.1E-08). Moreover, chromosomes 3, 7, and 20 are over-represented for SNP-mapped T2D protein-coding genes, with chromosome 20 still being most significant (P-value = 2.4E-06). The results suggest that chromosome 20 contains an unusually large number of T2D risk SNPs across diverse GWAS, and the risk SNPs can be mapped to a unusually large number of risk genes, particularly risk protein-coding genes, which cannot be simply attributed to inherent genotyping bias among GWAS across different human populations. The result suggests that chromosome 20 may be a risk chromosome.

**Table 1:** P-value of Chromosomal Specificity Significant Test.

Chromosome	P-value (SNP)	P-value (all genes)	P-value (coding genes)
1	5.3E-06	0.014	0.073
2	0.99	0.61	0.56
3	0.071	4.3E-03	0.011
4	0.99	0.21	0.12
5	1.00	0.88	0.81
6	0.039	0.47	0.58
7	0.087	0.037	0.023
8	1.00	0.70	0.60
9	1.00	0.96	0.95
10	0.15	0.38	0.38
11	2.8E-04	0.83	0.92
12	0.022	0.55	0.60
13	1.00	0.57	0.39
14	1.00	1.00	1.00
15	1.00	0.70	0.72
16	0.65	0.25	0.84
17	0.026	0.15	0.56
18	1.00	0.15	0.47
19	0.34	0.017	0.13
20	7.0E-38	6.1E-08	2.4E-06
21	0.94	0.63	0.58
22	0.98	1.00	1.00

Contributing to this result are genes including Hepatocyte Nuclear Factor 4 Alpha (HNF4A) and Protein Tyrosine Phosphotase non-receptor type 1 (PTPN1), both of which have been extensively studied for their roles to T2D genetics. Linkage studies dating back to 1997 showed a modest association with this region of chromosome 20 [26,27], when whole genome data was not available. Particularly interesting is that the interplay between genetics and environment also may act on this chromosomal region, e.g., the epigenetic effect of diet on the promoter regions of HNF4A [28].

### Strong network centrality in T2D high-risk genes

**Figure 1:**
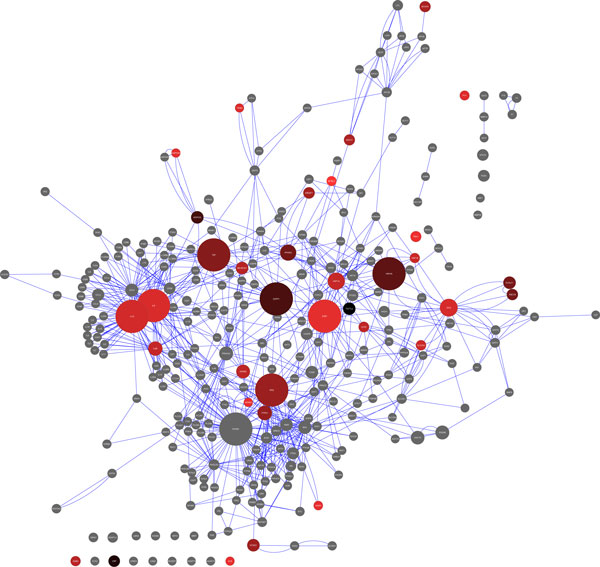
**T2D risk gene network**. Genes are represented as nodes drawn to the scale of their *r_mod _*scores. Higher-risk genes (high average odds ratio) are colored black and lower-risk genes (low average odds ratio) are colored red. Genes without significantly reported risk information are colored grey.

In Table 2, we show the original top-ranking T2D risk genes ordered by their *r_p _*scores, which we calculated based on T2D network connectivity information. After applying the filter "*r_p _*score > = 2" and re-constructing the T2D risk gene network, we derived a *r_mod _*score for each gene and show the network in Figure 1. The figure confirms that genes with high *r_mod _*scores are all network hubs with the resulting scores shown in Table 3. Genes with known *r_mod _*scores are shown to their node sizes and categorized by color. In Table 4, we listed top-ranking T2D genes ordered by *r_mod _*scores. Most of the top-5 ranking genes still kept their relatively high ranks before *r_mod _*score calculations.

**Table 2:** Top-ranking T2D risk genes ordered by their *r_p _*scores in the T2D risk gene protein interaction network.

Rank	Gene	*r_p _*Score
1	PI3KR1	114.09
2	ESR1	77.66
3	ENPP1	74.28
4	IL6	66.95
5	IL10	60.19
6	PRKAA2	56.47
7	PDE4B	56.33
8	ADCY3	53.45
9	IRS1	48.65
10	CD14	46.16
11	GCK	46.16
12	TCEB1	44.23
13	INSR	41.39

**Table 3:** Top-ranking T2D risk genes ordered by their *r_mod _*scores in the T2D risk gene protein interaction network.

Rank by *r_mod _*Score	Gene	*r_mod _*Score	*r_p _*Score	Original Rank by *r_p _*Score
1	ENPP1	430	74.3	3
2	ESR1	142	77.7	2
3	IL6	137	67.0	4
4	IL10	132	60.2	5
5	GCK	119	46.2	11
6	HNF4A	118	4.72	≫ 13
7	PIK3R1	114	-	1
8	TNF	110	3.75	≫ 13
9	IRS2	110	-	> 13
10	HNF1A	105	-	> 13
11	PPARG	82.9	4.27	≫ 13
12	PTPN1	69.8	3.69	≫ 13
13	IL6R	61.0	-	> 13

**Table 4:** Enriched pathways identified in the T2D risk genes.

Term	Count	%	P-value
Adipocytokine signalling pathway	15	7.01	6.21E-11
Type II diabetes mellitus	12	5.61	2.18E-09
Insulin signalling pathway	17	7.94	1.60E-08
Maturity onset diabetes of the young	8	3.74	5.63E-07
PPAR signalling pathway	9	4.21	9.11E-05
Calcium signalling pathway	10	4.67	1.20E-02
Renal cell carcinoma	6	2.80	1.60E-02
Hypertrophic cardiomyopathy (HCM)	6	2.80	3.38E-02
Aldosterone-regulated sodium reabsorption	4	1.87	5.55E-02
VEGF signalling pathway	5	2.34	7.43E-02
Adherens junction	5	2.34	8.02E-02
Pathways in cancer	12	5.61	8.68E-02

Our study suggests PI3KR1 may be the gene with highest T2D genetic risk associated. PIK3R1 is a regulatory subunit of the phosphoinositide-3-kinase, which is a protein known to be involved in insulin actions, cancer signaling, and cytokine signaling. While the coverage of the gene's functional relationship to T2D risks in T2DGADB is very limited (with one article only) [29], there is increasing evidence, including a recent SNP UTR study [30] and a mixed methods meta-analysis [31], that supports our finding.

The second highest-ranking gene in the T2D risk gene network by *r_p _*scores is ESR1, the estrogen receptor 1 gene. The gene encodes a transcription factor that responds to estrogen action and cancer, and will also form a heterodimer with ESR2. There are two articles cited in the T2DGADB database [32,33].

The third highest-ranking gene in the network is ENPP1, Ectonucleotide Pyrophosphatase/Phosphodiesterase 1, a trans-membrane glycoprotein involved in metabolism and has been shown to have an effect of insulin signaling and glucose metabolism [34]. ENPP1 was well studied among 20 GWAS-related publications collected through T2DGADB and 10 of those studies returned positive results in the population examined.

To demonstrate that the network hub genes are indeed functionally associated with T2D risks, we performed a t-test on the distribution of risk SNP per gene between the top ranked 25% of risk genes and the bottom 75% of risk genes. When ranks are given by the original *r_p _*scores, the results showed significant difference (P = 0.01) between the two groups for the reported risk SNP per gene raition (3.06 SNP/gene for the top 25% "hub genes" vs. 1.67 SNP/gene for the bottom 75% "non-hub genes"). When ranks are given by the modified *r_p _*scores, the results showed even higher significant difference (P = 3.55E-4) between the same two groups (3.74 SNP/gene for the top 25% "hub genes" vs. 1.45 SNP/gene for the bottom 75% "non-hub genes").

### Functional heterogeneity and cohesion of T2D high-risk genes

To gain an overview of functional categories represented by the T2D high-risk genes, we mapped these genes onto curated pathways. In Table 4, we list top significantly over-represented pathways identified by the DAVID software. Two highly ranked "novel" pathways identified (excluding known T2D pathways) are the Adipocytokine signaling and the PPAR signaling pathway.

**Figure 2:**
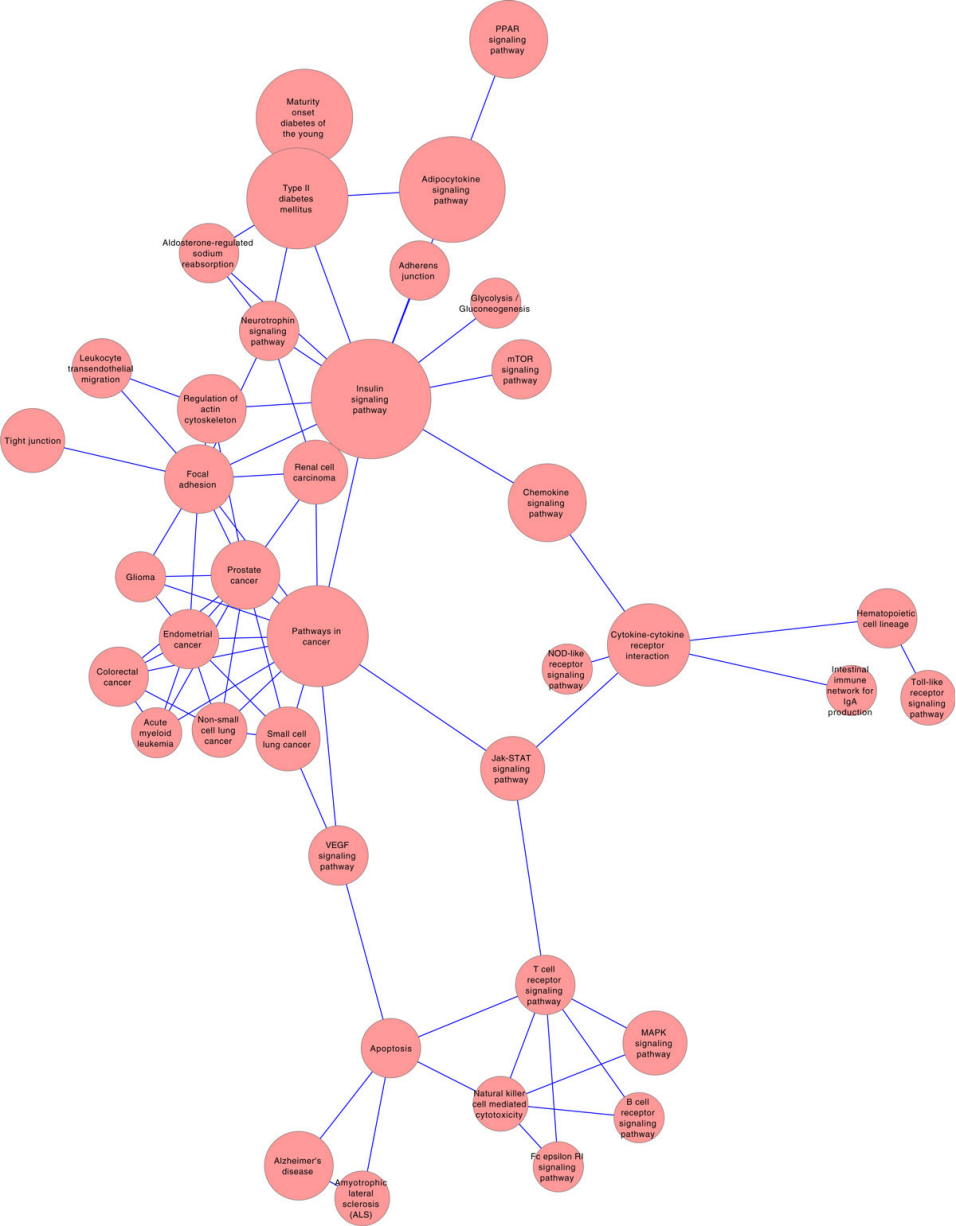
**T2D risk gene pathway interaction network**. Here, an edge will be created between two pathways, if and only if the pathways involved three of more risk genes.

From this result, we constructed T2D risk gene pathway interaction network (in Figure 2), if and only if interacting pathways involve 3 or more risk genes. The pathway interaction network showed distinct clusters involving cancer signaling. A few instead of many of identified risk genes may have contributed to the formation of these clusters. In the cancer pathway cluster, CASP9 and PIK3R1 are both involved in 7 different cancer pathways, while SOS1 and TCF7L2 are both involved in 5 different pathways.

**Figure 3:**
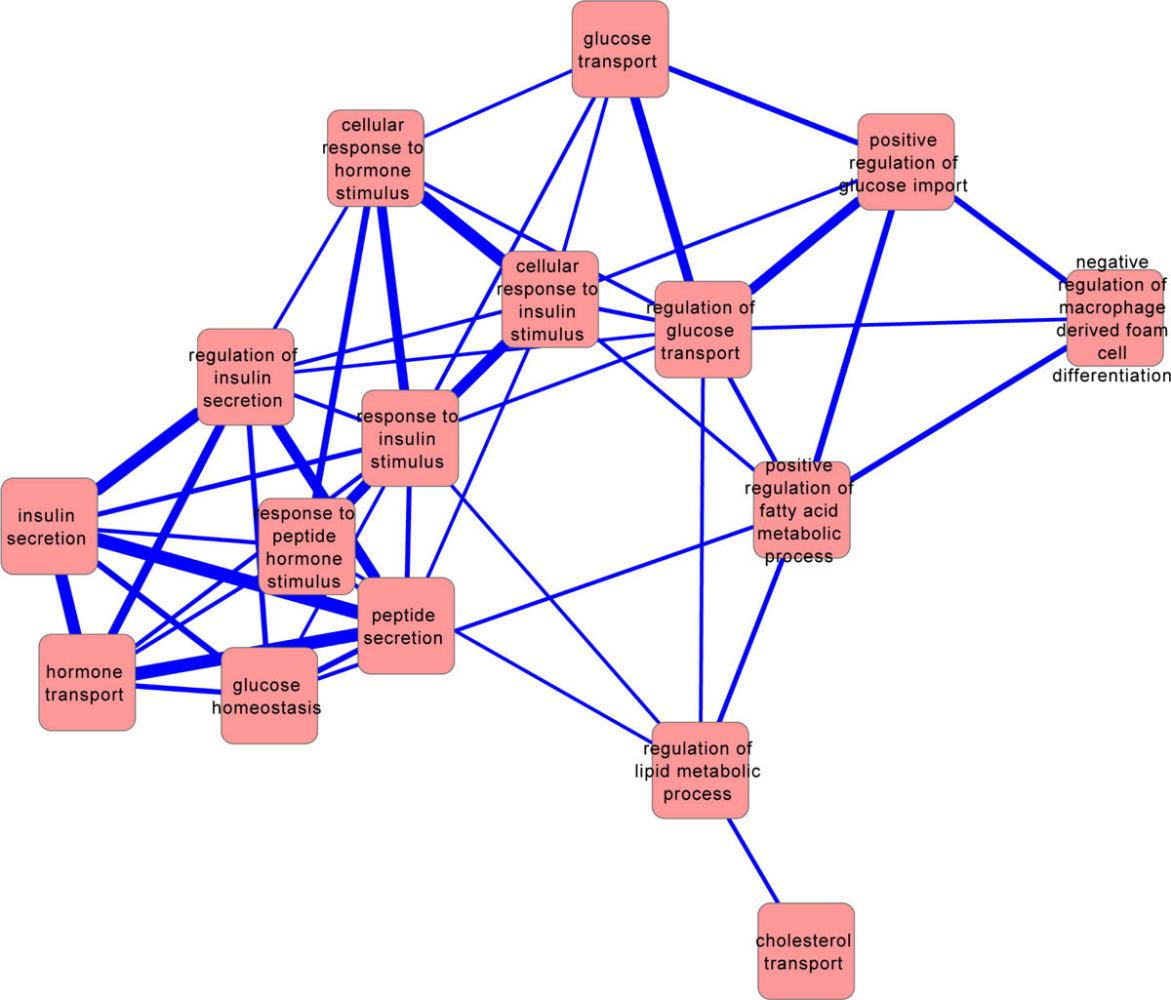
**T2D risk gene functional category crosstalk network**. For this figure an edge will be created between two functional categories for all significant Gene Ontology catagories.

Using the GARNET software, we also identified highly enriched gene ontology (GO) categories (as shown in Table 5) and "crosstalk" between GO functional categories (as shown in Figure 3). All these results confirmed the high relevance of T2D risk genes to glucose-related metabolism and insulin-related hormonal regulations. Pathway analysis even revealed possible activation of pathways related to cancer/cell cycle controls.

**Table 5:** Enriched gene ontology categories identified in the T2D risk genes.

Gene Ontology Term	Genes Involved	P Value
glucose homeostasis	16	6.16E-09
regulation of glucose transport	12	6.61E-08
regulation of insulin secretion	15	6.59E-08
glucose transport	15	3.33E-07
peptide secretion	19	2.82E-07
insulin secretion	17	3.78E-07
response to insulin stimulus	22	2.42E-06
response to peptide hormone stimulus	27	3.02E-06
cellular response to hormone stimulus	25	3.79E-06
hormone transport	20	6.62E-06
cholesterol transport	12	8.92E-06
positive regulation of glucose import	8	9.49E-06
positive regulation of fatty acid metabolic process	8	1.42E-06
cellular response to insulin stimulus	16	1.83E-05
negative regulation of macrophage derived foam cell differentiation	6	2.02E-05
positive regulation of glucose metabolic process	7	3.00E-05
regulation of lipid metabolic process	19	3.42E-05

## Discussion

In this study, we showed our findings of T2D genetic risk gens. Genes from the chromosome 20 collectively accounted for the highest T2D genetic risks of all the chromosomes. However, the individual contribution of these chromosome 20 risk genes is relatively small. HNF4A as the most significant gene on chromosome 20 has a relatively small *r_p _*score of 4.72, which is far lower than all *r_p _*scores shown for the top-ranking T2D risk genes in Table 2. Nonetheless, when all other information derived from GWAS results are integrated into the *r_mod _*score of 118, the significant contribution of HNF4A to T2D risks becomes clear. The "missing inheritability" problem of T2D genetic risks are therefore partially explained with the calculated integration of T2D molecular interaction network information and T2D genotype-phenotype association study results.

In this study, the findings depend on the quality of underlying data that we integrated from network biology, GWAS results, and SNP annotations. The data is complex, often derived from many different sources and groups. For example, TCF7L2, the most commonly studied gene in the collected data set, was covered by 45 GWAS, with up to a 3.4 odds ratio reported in a Finnish study [35]. However, such results face tremendous challenges in getting duplicated in other populations such as Japanese [36], Chinese [37], and Pima Indian [38]. In Table 6, we demonstrate the heterogeneity of results reported for the gene reported in different populations. Apparently, the gene is least significant in the "African American" population (average odds ratio is 1.19, among the lowest shown; yet it seems highly significant among Japanese and French, with average odds ratios being 1.57 and 1.62 respectively.

**Table 6:** GWAS results show population-specific effectiveness in using TCF7L2 for T2D genetic risk profiling.

Population	Number of Studies	Average Odds Ratio	Maximum Odds Ratio	Minimum Odds Ratio
American	7	1.36	2.14	0.82
Swedish	6	1.49	2.15	1.08
Finnish	5	1.45	3.40	1.01
UK	5	1.50	2.47	1.16
Japanese	3	1.57	2.08	1.18
French	2	1.62	1.84	1.45
African American	2	1.19	1.39	1.02
German	2	1.37	1.51	1.24
Dutch	2	1.47	1.96	1.29
Indian	2	1.58	2.28	1.29
American Indian	2	1.46	1.93	1.15

TCF7L2 was not among the highest ranked T2D genes, primarily due to the emphasis on the quality of protein interaction data that we bring in. The HAPPI database reported 238 protein interactions for TCF7L2 but only 11 of those were above the confidence threshold of 0.8. This is in contrast to ENPP1, in which we identified 743 protein interactions from the HAPPI database and 87 of them passed our confidence threshold of 0.8. The relatively low network centrality explained why TCF7L2 is not ranked at the top overall, although it is a population target for many T2D GWAS.

Future analysis that is built upon this work could benefit by integrating additional genomics and functional genomics information, e.g., available miRNA or mRNA expression information, available copy number variations results, and whole genome sequencing data.

## Conclusions

Large-scale systems biology meta-analyses of GWAS results can improve interpretations of genetic variations and genetic risk factors. In this work, we determined that T2D candidate risk genes are located in higher concentration in certain parts of the genome, specifically in chromosome 20. Using the T2D genetic network, we identified highly interconnected network "hub" genes. By incorporating T2D GWAS results, T2D pathways, and T2D genes' functional category information, we further ranked T2D risk genes, T2D-related pathways, and T2D-related functional categories. Overview maps involving T2D genes, gene sets, pathways, and their interactions are all reported. Moreover, we demonstrate a computational framework built upon disease-specific data integration, model construction, and data analysis. The framework developed for T2D studies may serve as a guide for studying other complex diseases.

## Competing interests

Jake Chen is the co-founder of Medeolinx, LLC, a company dedicated to providing bioinformatics solutions to pharmaceutical companies engaging in predictive and personalized medicine. Jake Chen declares no conflict of interest for this work.

## Authors' contributions

JYC conceived the idea and designed the analytical framework for this work. PJH implemented the design, collected data, and analysed results. Both JYC and AML provided timely guidance and critical feedback throughout the research. The manuscript was drafted by PJH and carefully revised by JYC and AML.
